# Volatilome, Microbial, and Sensory Profiles of Coffee Leaf and Coffee Leaf-Toasted Maté Kombuchas

**DOI:** 10.3390/foods13030484

**Published:** 2024-02-02

**Authors:** Amanda Luísa Sales, Sara C. Cunha, Isabel M.P.L.V.O. Ferreira, Jéssika Morgado, Lauro Melo, Juliana DePaula, Marco Antonio L. Miguel, Adriana Farah

**Affiliations:** 1 Núcleo de Pesquisa em Café Prof. Luiz Carlos Trugo (NUPECAFÉ), Laboratóriode Química e Bioatividade de Alimentos, Instituto de Nutrição, Universidade Federal do Rio de Janeiro, Avenida Carlos Chagas Filho, 373, CCS, Bl. J, Rio de Janeiro 21941-902, Brazil; amandasales@ufrj.br (A.L.S.); jessikarmorgado@gmail.com (J.M.); julianadepaula@nutricao.ufrj.br (J.D.); 2 Laboratório de Microbiologia de Alimentos, Instituto de Microbiologia Paulo de Góes, Universidade Federal do Rio de Janeiro, Avenida Carlos Chagas Filho, 373, CCS, Bl. I, Rio de Janeiro 21941-902, Brazil; 3 LAQV/REQUIMTE, Laboratório de Bromatologia e Hidrologia, Departamento de Ciências Químicas, Faculdade de Farmácia da Universidade do Porto, 4099-030 Porto, Portugal; sara.cunha@ff.up.pt (S.C.C.);; 4 Laboratório de Análise Sensorial e Estudos do Consumidor (LASEC), Escola de Química, Universidade Federal do Rio de Janeiro, Avenida Athos da Silveira Ramos, 149, CT, Bl. E, Rio de Janeiro 21941-909, Brazil; lauro@eq.ufrj.br

**Keywords:** coffee production waste, coffee by-product, coffee leaf, Rate-All-That-Apply, fermented beverages, yerba mate, toasted maté tea, volatile compounds, novel foods

## Abstract

Kombucha is a fermented beverage traditionally made from the leaves of *Camelia sinensis*. The market has drastically expanded recently, and the beverage has become more elaborated with new, healthy food materials and flavors. Pruning and harvesting during coffee production may generate tons of coffee leaves that are discarded although they contain substantial amounts of bioactive compounds, including those found in maté tea and coffee seeds. This study characterized the changes in volatilome, microbial, and sensory profiles of pure and blended arabica coffee leaf tea kombuchas between 3–9 days of fermentation. Acceptance was also evaluated by consumers from Rio de Janeiro (*n* = 103). Kombuchas (K) were prepared using black tea kombucha starter (BTKS) (10%), sucrose (10%), a symbiotic culture of Bacteria and Yeasts (SCOBY) (2.5%), and a pure coffee leaf infusion (CL) or a 50:50 blend with toasted maté infusion (CL-TM) at 2.5%. The RATA test was chosen for sensory profile characterization. One hundred volatile organic compounds were identified when all infusions and kombucha samples were considered. The potential impact compounds identified in CL K and CL-TM K were: methyl salicylate, benzaldehyde, hexanal, nonanal, pentadecanal, phenylethyl-alcohol, cedrol, 3,5-octadien-2-one, β-damascenone, α-ionone, β-ionone, acetic acid, caproic acid, octanoic acid, nonanoic acid, decanoic acid, isovaleric acid, linalool, (S)-dihydroactinidiolide, isoamyl alcohol, ethyl hexanoate, and geranyl acetone. Aroma and flavor descriptors with higher intensities in CL K included fruity, peach, sweet, and herbal, while CL-TM K included additional toasted mate notes. The highest mean acceptance score was given to CL-TM K and CL K on day 3 (6.6 and 6.4, respectively, on a nine-point scale). Arabica coffee leaf can be a co-product with similar fingerprinting to maté and black tea, which can be explored for the elaboration of potentially healthy fermented beverages in food industries.

## 1. Introduction

According to the Food and Agriculture Organization (FAO), sustainable food and agriculture is one in which food is nutritious and accessible to all and where natural resources are managed in a way that sustains ecosystem functions to meet present and future human needs [1]. The Sustainable Development Agenda for 2030 adopted by United Nations member States in 2015 and revised in 2021, was also adopted by FAO as a plan of action that provides a vision for a world free of extreme poverty and hunger, with reduced inequalities and a healthy natural environment through the transformation of developing more efficient, inclusive, resilient, and sustainable agrifood systems [2]. Agrifood wastes can be relevant sources of nutrients and bioactive compounds, providing multiple health benefits and adding value to the production chain.

Coffee is one of the most widely consumed beverages in the world and one of the most traded commodities globally, with worldwide production and overall consumption of approximately 10 million tons, from which *Coffea arabica* accounts for about 60–65% [3,4]. This market continues to grow for a variety of reasons, including increased consumption in emerging economies, interest in specialty coffee, and product innovations in developed countries [4]. Harvesting during coffee production generates coffee leaves as a byproduct, which are generally considered to be of no or low value as compared to the highly valuable coffee seeds, especially in organic crops. It has been estimated that about 3.3 tons of leaves are discarded per hectare during harvest season in Brazil because of the mechanical and stripping harvest methods practiced in mainstream coffee production [5]. Pruning is practiced in all crops worldwide, including specialty coffee. The discarded leaves can be used to produce various bioproducts, adding value to coffee production.

Coffee leaves have been used for tea production for centuries, particularly in Africa and Asia [6,7]. They contain several phytochemicals such as chlorogenic acids, mangiferin, isomangiferin, rutin, quercetin, theobromine, caffeine, and trigonelline, terpenes, and other compounds that are known to provide antioxidant and anti-inflammatory effects and promote benefits such as antihypertensive and immunomodulatory effects, and relief of gastrointestinal symptoms, among others [6,7,8,9,10]. The consumption of coffee leaf tea is growing in Europe and is expected to continue to grow, replacing or complementing coffee or other tea beverages in many homes [11].

Fermented foods have been staples of the human diet for centuries and are an increasingly popular food category [12]. Fermented beverages are on the rise worldwide, growing rapidly and attracting the most health-conscious consumers.

A preliminary sensory analysis revealed that the fermentation of coffee leaves by *Saccharomyces cerevisiae* and *Latilactobacillus plantarum* produced promising coffee leaf teas among European consumers [13]. Blending coffee leaf with toasted maté was considered as another alternative for introducing coffee leaf tea to Brazilian consumers [14], given the high and frequent consumption of maté tea in Brazil, including in Rio de Janeiro where it is drunk toasted and cold [15].

Kombucha is traditionally produced through the fermentation of *Camellia sinensis* teas and sugar in a symbiotic association of bacteria and yeasts called SCOBY. It originated in Asia in around 220 B.C. and was first used for its health properties [16]. The traditional beverage can be prepared using both black and green teas, with the former being more popular [17]. Kombucha has numerous benefits supported by in vitro and in vivo studies, such as antioxidant and anti-inflammatory activities [18,19,20]; antiproliferative activity in colon, breast, and lung cancer cells [20,21,22]; antibacterial activity [21]; hypoglycemic effects; and weight loss in diabetic rats [23].

Praised for its digestive benefits, kombucha proliferates in retail, taking up more shelf space in health specialist stores and traditional grocery stores [24]. In 2022, the global market value of kombucha was estimated at over two billion U.S. dollars. By 2028 the market is expected to reach over six billion U.S. dollars [25]. The growing interest in kombucha has attracted the use of new raw materials for fermentation that are different from black tea, such as teas, fruits, herbs, and milk, as well as a wide range of agro-industrial materials, which are mainly byproducts of the fruit industry [26].

A limited number of studies have been performed on the potential use of coffee leaves for the elaboration of food products [27]. Considering the taste, bioactive profile, and market potential of coffee leaf tea, the similarities between *Camelia sinensis* leaves and *Coffea* leaves, and the market potential of fermented beverages, elaborating a kombucha beverage from coffee leaf tea seems to be a promising idea for promoting consumer health, sustainability, and the value of coffee production. Therefore, the aim of this study was to characterize the changes in volatilome, microbial, and sensory profiles of kombuchas made with arabica coffee leaf tea, pure and blended with toasted maté, for 3–9 days of fermentation. Acceptance was also evaluated by consumers from Rio de Janeiro.

## 2. Materials and Methods

### 2.1. Samples

All pure tea samples were obtained by combining multiple sample units to form a composite sample of each pure tea. The following samples were used to prepare the infusions for kombucha fermentation: the leading commercial brand of unfermented *Coffea arabica* leaf tea—CL in the West, harvested and processed in Nicaragua and sold in Canada; the leading commercial brand of black tea (*Camellia sinensis*)—BT in Brazil; and the leading brand of commercial toasted maté (*Ilex paraguariensis*)—TM in Rio de Janeiro, Brazil [28]. A blend was also prepared with 50% CL and 50% TM. All leaves were used in bulk.

### 2.2. Kombucha Consortium and Microbial Analysis

The kombucha consortium was obtained from the Microbiology Institute of the Federal University of Rio de Janeiro in Brazil. In order to stabilize the microbial consortium originally grown in green tea, three separate fermentation processes in black tea, coffee leaves or coffee leaves with maté infusions were performed before experimental use [29]. DNA was extracted from samples of SCOBY and samples of kombuchas that have been fermented for 9 days, according to Yamanaka et al. [30] method adapted by Sales et al. [19].

### 2.3. Kombucha Preparation

The kombucha preparation protocol was adapted from Nummer [31].

*Infusions*: Infusions of BT were prepared at a concentration of 3%, while infusions of CL and CL-TM were prepared at a concentration of 2.5% (*w*/*v*), based on preliminary sensory results. Water at 95 °C was poured over the raw material and allowed to steep for 10 min. The leaves were then removed using a traditional sieve for infusion preparation. *BT kombucha starter*: BT kombucha starter was prepared by mixing 90% (*v*/*v*) BT infusion, 10% (*w*/*v*) sugar, 10% (*v*/*v*) BT K previously fermented (starter culture), and 2.5% (*w*/*v*) of the SCOBY and was fermented at 23 °C for 9 days to be used as a starter (pH = 2.8 ± 0.05) for the other beverages. After fermentation, samples were collected for Volatile Organic Compound (VOC) analyses.
*CL or CL-TM kombucha*: CL kombucha beverages (CL K) were prepared by mixing 90% (*v*/*v*) CL or CL-TM infusion, 10% (*w*/*v*) sugar, 10% (*v*/*v*) BT K starter culture, and 2.5% (*w*/*v*) of SCOBY and fermented at 23 °C. Sampling was done every 3 days during the 9 days of fermentation (d0, d3, d6 and d9) After fermentation, the samples were collected for analyses of Volatile Organic Compounds (VOCs).

### 2.4. pH, Total Titratable Acidity, Total Soluble Solids, and Sugars Analysis

pH was measured using a pH meter (Kasvi K39-0014PA, São José dos Pinhais, Paraná, Brasil). The total titratable acidity was determined by titration with 0.1 N NaOH and phenolphtalein as the indicator, according to the Adolfo Lutz Institute [32]. The results were expressed in mEq/L. The total soluble solids were evaluated using a handheld refractometer (Pocket Refractometer Pal-1, ATAGO, Tokyo, Japan). The results were expressed in °Brix.

Sucrose was analyzed using a High-Performance Liquid Chromatography Refractive Index Detector (RID) system (mod.# 2414, Waters, Milford, MA, USA), according to Wischral et al. [33] as in Sales et al. [19]. External standards calibration curves were used to identify and quantify all sugars.

### 2.5. Analyses of Volatile Organic Compounds

The extraction of VOC from the infusions and kombuchas was performed by headspace solid-phase microextraction (HS-SPME) using a 50/30 μm divinylbenzene/carboxen/polydimethylsiloxane fiber (DVB/CAR/PDMS, Supelco^®^, Bellefonte, PA, USA). Analysis was performed by a gas chromatographer (Agilent, 6890 Little Falls, DE, USA) coupled to a mass spectrometer (Agilent 5975) (GC-MS), according to the methodology described by Wang et al. [34] and adopted by Sales et al. [35] and DePaula et al. [14].

### 2.6. Sensory Tests

The Ethical Committee of the Clementino Fraga Filho University Hospital at the Federal University of Rio de Janeiro (UFRJ) approved this study (# 4.513.606). The subjects, including students, teachers, visitors, and employees at the UFRJ Health Sciences and Technology Centers living in different areas of Rio de Janeiro provided written consent after being thoroughly informed. The eligible criteria for this study included habitual consumers of kombucha or sparkling beverages, such as sparkling water, ciders, and soft drinks. Individuals who had a positive COVID-19 diagnosis and experienced loss of taste and/or smell were excluded from the study, as were individuals with any other conditions that could affect sensory evaluation. A total of 103 participants took part in the Acceptance, Purchasing Intent, and Rate All That Apply (RATA) tests after exclusions.

Consumer assessors performed the tests on individual benches in the UFRJ Food and Dietetics Lab according to Sales et al. [35].

#### 2.6.1. Consumer Acceptance and Purchase Intent

The assessors used a nine-point hedonic scale (ranging from one, which means extremely disliked, to nine, which means extremely liked) to evaluate the infusions. This was followed by a five-point purchase intent scale (ranging from one, which means certainly would not buy, to five, which means certainly would buy) [36]. To calculate the Acceptability Index (AI) the following equation was used:

AI = (X × 100)/N

where X = Average score given by assessors and N = Highest score given by assessors. An AI equal to or greater than 70% was considered satisfactory [36].

#### 2.6.2. Rate All That Apply (RATA)

The assessors were provided with a pre-prepared checklist of 34 sensory descriptors related to appearance, aroma, flavor, and mouthfeel. These descriptors were identified in a preliminary session by a trained panel of nine experts (aged 28–58) with a minimum of 200 h of experience in evaluating different food products and 30 h of experience in evaluating fermented beverages and infusions. The panelists were asked to generate their individual descriptors using a modified grid method [37]. Afterward, they agreed on the best descriptors for fully describing the samples and the evaluation methods [38]. The sensory descriptors used in the study were organized by alphabetical order as follows: burnt, fermented, fruity, green leaf, herbal, peach, rosé wine, sweet, toasted leaf, white wine (for odor); acidic/sour, bitter, and sweet (for taste); acetic/vinegar, alcoholic, apple vinegar, fruit syrup, fruity, toasted leaf, green apple, green coffee, herbal, peach, white wine, and brewer’s yeast (for flavor); astringency, sparkling, fizzy, full-bodied, refreshing, and watery (for mouthfeel); and clear, brown, and opaque/matte (for appearance). To determine whether assessors perceived the varying intensities in the aroma, taste, and flavor descriptors of the kombucha samples, assessors were asked to score the descriptor based on their intensity using RATA scores (1 = low intensity, 2 = medium intensity, and 3 = high intensity).

### 2.7. Statistical Analysis

Data from physicochemical analyses are presented as mean ± standard deviation. One-way ANOVA, followed by the Tukey test, was performed to identify significant differences (GraphPad Prism, Version 8.4.2, Informer Technologies, Los Angeles, CA, USA).

Statistical analyses of VOC data was performed by Principal Component Analysis (PCA) using individual peak areas as variables (R version 4.2.2, RStudio team 2022, Boston, MA, USA). Data pretreatment included normalization and scaling, which are required processes for data that present wide-scale differences, as is the case for volatiles.

For sensory tests, statistical analyses were conducted using the XLSTAT software, version 2023.1.1 (Addinsoft, Paris, France). ANOVA followed by the Tukey test was performed for acceptance (fixed factors: fermentation time and maté addition; random factor: consumers; and interaction between fermentation time and maté addition) and purchase intent tests results. For RATA descriptors, ANOVA, followed by Fisher’s test and correspondence analysis based on chi-squared distances, was performed to achieve a sensory map of the samples [39]. The test of independence between rows and columns was carried out at 5% significance. Cluster analysis (agglomerative hierarchical clustering using Euclidean distance for the Ward method) was also applied [40,41]. Differences were considered significant when *p* ≤ 0.05.

## 3. Results and Discussion

### 3.1. Physicochemical Parameters

Table 1 presents the pH, total acidity, soluble solids, and sucrose values for all evaluated kombuchas. In CL K, the total acidity increased up to d9, and the pH decreased from 3.8/3.9 to 3.4. These values are within the ranges observed in the literature for kombuchas [22,42] and are caused by the fermentation process that forms several organic acids, which are mainly acetic, glucuronic, lactic, and citric acids, and the monosaccharides glucose and fructose [43,44]. The addition of TM to CL made the fermentation process slower; therefore, the pH decreased mildly from d0 to d9, which is in agreement with the literature [45].

The pH values are within the kombucha pH range considered safe for human consumption (2.5 to 4.2) [31]. Kombucha pH values below 2.5 have a high concentration of acetic acid, posing a risk to consumers’ health. Likewise, pH values above 4.2 may compromise the beverage’s microbiological safety [22].

Also, the kombucha pH has been shown by Ulusoy and Tamer [45] to stabilize due to the buffer effect caused by the organic acids and carbon dioxide formed during fermentation. According to the authors, the resulting aqueous solution of carbon dioxide dissociates and produces the amphiprotic hydrocarbonate anion (HCO_3_^−^), which quickly reacts with hydrogen ions (H^+^) from organic acids, preventing further changes in the (H^+^) concentration and contributing to the buffering character of the system.

The final average sucrose content in all kombuchas tended to decrease (37% in CL and 32.7% in CL-TM, from d0 to d9). Fermentation also decreased the soluble solids concentration, probably because of the decrease in the sucrose concentration in the culture medium over time [35,46]. At the beginning of the kombucha fermentation process, yeast produces invertase, which cleaves the disaccharide sucrose to its monosaccharide components: glucose, and fructose [44].

### 3.2. Microbial Taxonomy

**Figure 1:**
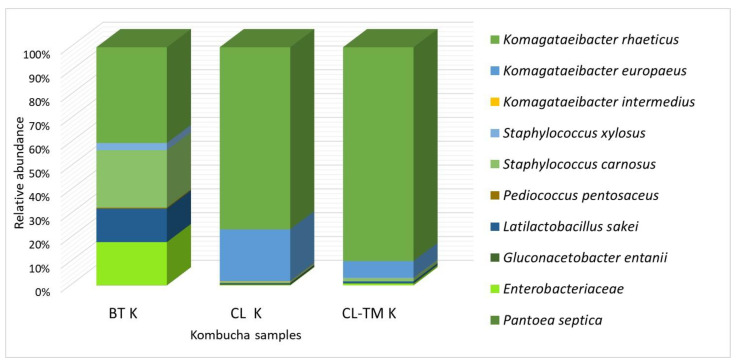
Characterization of the bacterial composition of the black tea kombucha starter (BT K) and that of coffee leaf (CL K) and coffee-leaf-toasted maté (CL-TM K) kombucha consortia after 9 days of fermentation.

**Figure 2:**
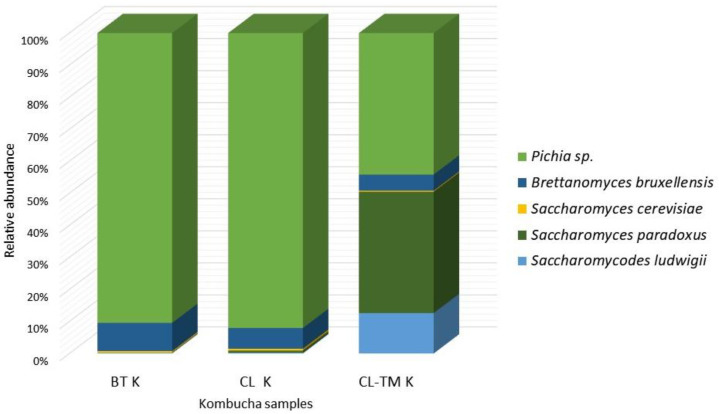
Yeast composition of the black tea kombucha starter (BT K) and the coffee leaf (CL K) and coffee-leaf-toasted maté (CL-TM K) kombucha consortia after 9 days of fermentation.

Figure 1 and Figure 2 characterize the microbial community of the SCOBY and the final kombuchas CL K and CL-TM K (d9). The SCOBY composition was similar to that which was reported by the same authors for coffee cascara kombucha [35], with slight differences in microorganism strains and percentages in the CL and CL-TM kombuchas. All samples were found to contain two bacterial phyla, Proteobacteria and Firmicutes, according to the data analysis of the 16S rRNA gene sequence (Figure 1). Proteobacteria was the overwhelmingly dominant phylum, with a percentage exceeding 90%, particularly in CL K and CL-TM K. This result is consistent with previous studies evaluating kombucha beverages’ microbial profile [47,48,49].

*Komagataeibacter*, the most efficient bacterial cellulose producer [50], was the most abundant genus found in both the liquid and biofilm of all kombuchas, which is consistent with previous studies characterizing kombucha cultures [51]. Only *Komagataeibacter rhaeticus* was identified in the starter culture, which accounts for about 40% of the total number of bacteria. This bacterium is known to be one of the most abundant members among kombucha fermenting agents [29,51]. In kombucha, *Komagateibacter* genera are positively correlated with the presence of furfural and benzaldehyde, among other volatile compounds, and less correlated with acetic acid and octanoic acid [52].

In CL K, characterized for the first time, *K. rhaeticus* comprised more than 70% of CL K microorganisms and about 90% of CL-TM K microorganisms contained in the liquid and solid cultures. In addition to a high percentage of *K. rhaeticus* (70–90%), *K. europaeus* (7–22%), *K. intermedius* (0.3%), and *Gluconacetobacter entanii* (0.5%) were identified in CL and CL-TM K. *K. europaeus* and *K. intermedius* have previously been identified in black tea kombuchas [29,53,54] and in coffee cascara kombucha [35]. According to Yao et al. [55], *K. europaeus* and *K.rhaeticus* are positively associated with acid production in kombucha flavor.

The *Gluconacetobacter* genus has been detected in black tea kombuchas [29,49], as well as in other fermented matrices [56] and kombuchas [57]. This genus possess valuable characteristics that can be combined with yeast strains for glucuronic acid production [58].

Low percentages of *Latilactobacillus* (0.3–0.8%), *Enterobacteriaceae* (0.2–0.8%), and *Staphylococcus* (0.4–1.3%) were observed. Additionally, two lactic acid bacteria, *Latilactobacillus sakei* and *Pediococcus pentosaceus*, were identified in BT K, CL K, and CL-TM K. It is worth noting that *L. sakei* was found to produce volatile compounds such as hexanal, acetic acid, and geranyl acetone in a model kimchi [59,60,61,62]. *Pediococcus pentosaceus* was confidently chosen to ferment tilapia surimi, resulting in the production of several key aldehydes such as hexanal, nonanal, heptanal, octanal, decanal, undecanal, and benzaldehyde [60,61,62,63], and was only identified in CL-TM K at a low percentage (0.02%).

*Staphylococcus carnosus* and *Staphylococcus xylosus* were identified in BT K, CL K, and CL-TM K. It has also been in coffee cascara kombuchas [35]. They are found in several fermented food products and are recognized as non-infective, contributing to acidic and buttery characteristics [61].

As also observed in coffee cascara kombucha, Enterobacteria were identified in CL K and CL-TM K, but in a lower percentage than in black tea kombuchas [35]. Enterobacteria are among commonly isolated microbial groups from spontaneous food fermentations, including kombucha fermentation [29,47,49]. *Pantoea septica* was an additional Enterobacteriaceae strain identified in the starter kombucha [63]. The *Pantoea* spp. genus was previously identified in grape cultivars for wine production and was positively correlated with straight-chain fatty alcohols, aromatic aldehydes, and terpenes in wine [64].

The most abundant yeast phyla was Ascomycota (Figure 2). *Pichia* was the most abundant yeast genera (>70%), followed by *Saccharomyces* (>20%) and *Brettanomyces* (5–8%) genera.

*Pichia* strains were previously identified as the main yeast genera in kombuchas [47,49,54]. *Pichia* strains identified in CL and CL-TM kombuchas were *Pichia fermentans, Pichia barkeri*, and *Pichia dianae*, which are different from coffee cascara kombucha, in which the strains *Pichia fermentans* and *Pichia kluyveri* were identified [35].

*Brettanomyces bruxellensis* was the most common yeast identified in black tea kombucha [29,51,64] and in coffee cascara kombucha [35]. Ester production during the fermentation is performed by the esterases present in *Brettanomyces* spp., which are responsible for the formation of ethyl esters, such as ethyl acetate and ethyl lactate, along with the hydrolysis of acetate esters, such as isoamyl acetate and phenethyl acetate, although this strain can also produce negative descriptors in fermentation products [65]. In kombucha, *Brettanomyces bruxelensis* can contribute to alcohols and acids production, such as isoamyl alcohol and phenylehtyl alcohol [66] and is positively associated with the acetic acid formation in kombucha [55].

The percentage of *Saccharomyces* sp. was similar to that observed by Landis et al. [51] but was lower than coffee cascara kombucha [35]. These genera and strains have been previously identified in kombuchas [47,49,55]. *Saccharomyces* sp. is the major yeast genus involved in producing alcoholic beverages [67]. *Saccharomyces* sp. strains have been previously identified in kombuchas [47,49,55]. During fermentation, *Saccharomyces cerevisiae* (0.4–0.5%) presented a higher abundance in CL-TM K. In kombucha, this strain is correlated with ethanol [53,56]. Another *Saccharomyces* strain identified was *Saccharomyces paradoxus*. This strain can produce high concentrations of hexanol, isoamyl alcohol, 2-phenylethyl ethanol, and ethyl acetate, among other volatile compounds, in wine [68].

*Saccharomycodes ludgwigii* is considered detrimental to the winemaking process [69,70]. This strain has been previously identified in coffee cascara kombucha [35].

### 3.3. Volatile Organic Compounds

**Figure 3:**
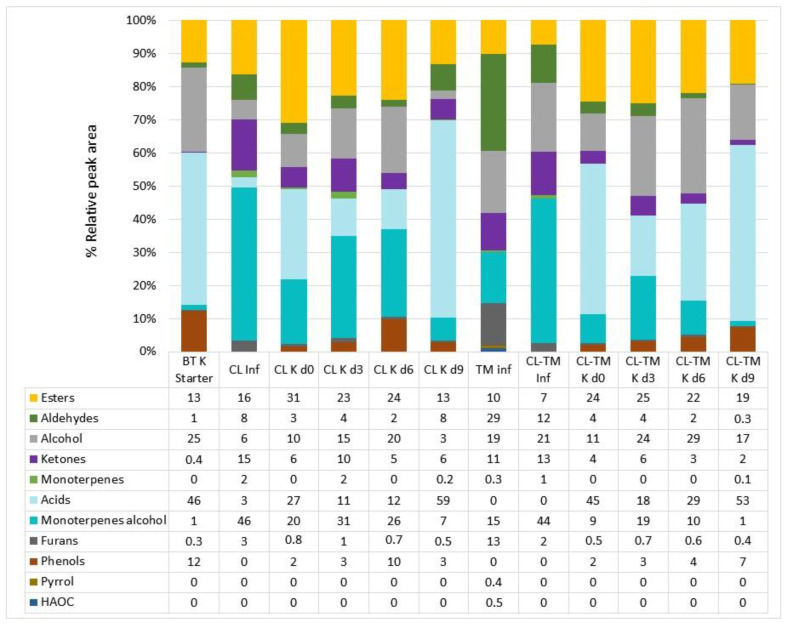
Relative peak areas (%) of volatile organic compounds in black tea kombucha starter (BT K), coffee leaf (CL K), and coffee leaf-toasted maté (CL-TM K) kombucha beverages grouped into chemical classes. Note.; Inf: infusion; d0, d3, d6, and d9: days of fermentation; HAOC: heterocyclic aromatic organic compounds.

Figure 3 presents the relative peak areas of volatile organic compounds (VOCs) (grouped by classes) in infusions and kombuchas made from CL, TM, and CL-TM. We also evaluated the VOCs from BT K starter culture, given that all kombucha beverages contained 10% of it. Although the area does not directly reflect the concentration of the compound, it serves an indicator of its relative abundance and, together with the total areas of the VOCs, provides the volatile profile, which is useful for comparison purposes [14]. The potential impact of the compounds will be presented later in this section.

The highest peak areas in BT K starter were from acids, alcohols, and esters due to its advanced fermentation stage. Generally, the infusions tended to present higher area percentages of aldehydes, ketones, monoterpene-alcohols, and furans compared to kombuchas, while the kombuchas presented more acids, esters, and phenols due to the fermentation process [71]. The substantial content of alcohols at d0 of fermentation in both kombuchas is derived from CL and TM raw materials and the starter culture [72,73].

For acids, the peak areas tended to increase with fermentation. Volatile acids are produced during alcoholic and acetic fermentation by the symbiosis between acetic acid bacteria and yeasts in SCOBY [73]. The starter culture contributes greatly to the percentage and number of acids found in kombuchas [73]. The high peak area of monoterpene alcohols could be explained by the free odor-producing forms of monoterpene alcohols, given the presence of glycosidically bound monoterpene alcohols in tea. Another possibility is the release of some aroma constituents when the non-volatile materials from tea leaves were fermented [74].

Fermentation products result from several complex and changeable enzymatic and/or chemical reactions involving volatile and nonvolatile precursors. These chemical changes result in the development of aroma and flavor. While BT, CL, TM, and CL-TM infusions can be characterized as having herbal/green leaf and woody aroma and flavor [14], kombucha production develops richer fruity and sometimes flowery aromas and flavors [75]. Common metabolic pathways include those of carbohydrates, amino acids, fatty acids, and other lipidic components, among other classes. These pathways interact and intertwine to shape the unique flavor of foods [76].

The VOCs identified in the BT K starter and the CL, TM, and CL-TM infusions and kombuchas are presented in Table 2. A total of 100 VOCs were identified, considering all beverages, among them 23 esters, 19 aldehydes, 13 alcohols, 12 ketones, nine monoterpenes, eight acids, five monoterpenes alcohols, five furans, two phenols, one pyrrol, and one heterocyclic aromatic compound. In the infusions, 37 VOCs were identified in CL, 48 in TM, and 67 in CL-TM.

Twenty compounds were identified only in the CL infusion and not in TM infusion: nonanoic acid, α-terpineol, and isopropyl palmitate were reported as odor-active compounds in coffee leaf tea [13]; methyl palmitate and 2-methylbutyraldehyde were identified in black tea [77]; the monoterpenes myrcene, 3-δ-carene, and camphene were significant contributors for green, black, and Pu-erh teas [78,79,80,81,82]; Z-3-hexenol and (S)-2-methyl-1-butanol were identified in green tea [81]; cedrol was identified in white tea [83]; 1-terpinen-4-ol was a key component of citrus fruit aroma [84]; and additional compounds included dodecanal, acetic acid, ethyl palmitate, linalyl acetate, (−)-carvone, tetradecanal, 2-methylbutanoic acid, and phenethyl alcohol.

CL and TM infusions showed similar VOC profiles and 16 compounds, among which were nine potential odor-impact compounds: α-ionone, ß-ionone, 3,5-octadien-2-one hexanal, nonanal, benzaldehyde, trans-linalool oxide, linalool, and dihydroactinidiolide [13,85,86,87,88]. Their odor descriptions are listed in Table 2. They will also be discussed in the kombucha section.

Twenty-nine VOCs were only identified in the TM infusion and not in the CL. Among them, six potential impact compounds were identified: 2,4-heptadienal, nonanal, safranal, ethanol, geranyl acetone, and β-damascenone [13,85,86,87,88]. Among the additional compounds were the carotenoid-derived dihydro-β-ionone and megastigmatrienone, which are characteristic of tobacco aroma [89,90]. The heating process (drying and scorching), to which the toasted maté leaves are subjected before consumption, leads to many degradation products and enhances the flavor resembling tobacco [86]. In this study, CL was only dried, not scorched and dried like TM. Regarding the toasted maté compounds, the ketone ketoisophorone, which contributes to woody and aged aromas in *C. sinensis* An tea [91], is also a freshness marker in saffron [92], while 2,3-butanedione was previously identified in watermelon juice fermented by lactic acid bacteria [93]. The aldehyde benzeneacetaldehyde is an impact compound in oolong tea [94]. Furans and pyrroles are heat treatment markers [95]. These VOCs can be generated by the Maillard reaction during *C. sinensis* tea manufacturing [96] and also during coffee roasting [97]. Furfural, 5-methylfurfural, 2-ethylfuran, and 1-furfurulpyrrole have been previously identified in toasted maté infusions [14]. Another volatile Maillard reaction product is 4,5-Dimethyl-2-propyloxazole [98]. Additional compounds identified only in TM were benzophenone, 2,6,6-trimethylcyclohexanone, phtalolactone, 2-heptenal, 1-penten-3-ol, p-mentha-1,5,8-triene, and 2-methylallyl butyrate. These compounds were identified in toasted maté by DePaula et al. [14].

When kombuchas were obtained, an increase in the number of VOCs was observed, from 37 to 75 in CL, with 24 derived from the BT K starter and 13 formed during fermentation. Of these newly formed compounds, 54% were esters and 23% were aldehydes. In CL-TM, the number of identified compounds increased from 67 to 90 after fermentation. Of these compounds, 20 were from the BTK starter, and seven were formed during fermentation, with 100% being esters. A similar increase in the number of VOCs was reported for coffee cascara kombucha [35].

**Table 2:** Volatile compounds identified in coffee leaf and coffee-toasted maté leaf kombuchas.

Volatile Compound	Odor Description	CAS# ^a^	ELRI ^b^	LRI ^c^	BT K Starter	CL Inf	CL d0	CL d3	CL d6	CL d9	TM Inf	CL-TM Inf	CL-TM d0	CL-TM d3	CL-TM d6	CL-TM d9
**Esters**																
2-Methylbutyl acetate	Fruit, over ripe fruit, sweet, banana, juicy ^1,2^	624-41-9	677	848	■	□	■	□	□	□	□	□	■	■	■	■
2-Methylallyl butyrate	Powerful, fruity, ether, sweet, pineapple, apple, plum ^2^	7149-29-3	696	774	□	□	□	□	□	□	■	■	■	□	□	□
Ethyl acetate	Pineapple, ethereal, fruity, sweet, weedy, green ^1,2^	141-78-6	971	977	■	□	■	■	■	■	□	□	■	■	■	■
Ethyl phenylacetate	Fruit, sweet, floral, honey, rose, balsam, cocoa ^1,2^	101-97-3	877	904	□	□	□	□	■	■	□	□	□	□	■	■
Ethyl butyrate	Apple, fruity, juicy, pineapple, cognac ^1,2^	105-54-4	765	848	□	□	□	□	□	□	□	□	□	□	□	■
Ethyl isobutyrate *	Sweet, rubber, ethereal, fruity, alcoholic, fusel, rummy ^1,2^	97-62-1	882	895	■	□	■	■	■	■	□	□	■	■	■	■
Ethyl 2-methylbutyrate	Sharp, sweet, green, apple, fruity ^2^	7452-79-1	899	947	■	□	■	■	■	■	□	□	■	■	■	■
Ethyl 3-hexenoate	Fruity, pineapple, green, tart, candy, metallic, tropical, rhubarb, weedy, cheesy ^2^	2396-83-0	909	920	□	□	□	□	■	■	□	□	□	□	□	□
Ethyl (Z)-3-hexenoate	Green, pear, apple, tropical ^2^	64187-83-3	914	933	□	□	□	□	□	□	□	□	□	□	□	■
Ethyl hexanoate *	Apple peel, fruit, sweet, pineapple, waxy, green, banana ^1,2^	123-66-0	921	924	□	□	□	□	■	■	□	□	□	□	■	■
Ethyl valerate	Yeast, fruit, sweet, apple, pineapple, green, tropical ^1,2^	539-82-2	798	876	□	□	□	□	■	■	□	□	□	□	■	■
Ethyl octanoate	Fruit, banana, pear^1,2^	106-32-1	876	899	■	□	■	■	■	■	□	□	■	■	■	■
Ethyl nonanoate	Fruity, rose, waxy, rum, wine, natural, tropical ^2^	123-29-5	876	899	□	□	□	□	□	□	□	□	□	□	■	■
Ethyl decanoate	Grape, sweet, waxy, fruity, apple, oily, brandy ^1,2^	110-38-3	913	928	■	□	■	■	■	■	□	□	■	■	■	■
Ethyl laurate	Leaf, sweet, waxy, floral, soapy, clean ^1,2^	106-33-2	843	880	■	□	■	■	■	■	□	□	■	□	□	□
Ethyl palmitate	Wax, fruity, creamy, milky, balsamic, greasy, oily ^1,2^	628-97-7	739	820	□	■	■	□	□	□	□	■	■	□	□	□
Isoamyl acetate *	Banana, sweet, fruity, solvent ^1,2^	123-92-2	759	859	□	□	□	□	■	■	□	□	□	□	■	■
Linalyl acetate	Sweet, fruit, green, citrus, bergamot, lavender, woody ^1,2^	115-95-7	772	809	□	■	■	□	□	□	□	■	■	□	□	□
Isopropyl myristate	Faint, oily, fatty ^2^	110-27-0	856	867	□	■	■	□	□	□	■	■	■	□	□	□
Isopropyl palmitate	Fat, bland, oily ^1,2^	142-91-6	826	894	□	■	■	■	■	□	□	■	■	□	□	□
Methyl salicylate *	Peppermint ^1^	119-36-8	889	928	□	■	■	■	■	■	■	■	■	■	■	■
Methyl dihydrojasmonate	Floral, oily, jasmin, green, lactonic, tropical, natural ^2^	24851-98-7	785	823	□	□	□	■	■	■	□	□	□	□	□	□
Methyl palmitate	Oily, waxy, fatty, orris ^2^	112-39-0	747	798	□	■	■	□	□	□	□	■	■	□	□	□
**Aldehydes**																
Ethanal/Acetaldehyde	Pungent, ether, fresh, fruity, musty ^1,2^	75-07-0	976	976	■	□	■	■	■	■	■	■	■	■	■	■
2- Methylbutyraldehyde	Musty, cocoa, phenolic, coffee, nutty, malty, fermented, fatty, alcoholic ^2^	96-17-3	644	754	□	■	■	■	□	□	□	■	■	□	□	□
Benzaldehyde *	Almond, burnt sugar, tropical fruit ^1,2^	100-52-7	736	885	□	■	■	■	■	■	■	■	■	■	■	■
Benzeneacetaldehyde	Honey, floral, sweet, fermented, chocolate, earthy, green ^1,2^	122-78-1	904	961	□	□	□	□	□	□	■	■	■	□	□	□
Phenylacetaldehyde	Honey, floral, rose, sweet, powdery, fermented, chocolate, earthy, hawthorne, green, hyacinth, clover, cocoa ^1,2^	122-78-1	932	954	□	■	■	■	■	■	■	■	■	■	■	■
Isovaleraldehyde	Ethereal, aldehydic, chocolate, peach, fatty ^2^	590-86-3	777	842	□	■	■	■	□	□	■	■	■	□	□	□
Hexanal *	Grass, tallow, fat, fresh, green, aldehydic, leafy, fruity, sweaty ^1,2^	66-25-1	800	855	□	■	■	■	■	■	■	■	■	■	■	■
(E)-2-Heptenal	Pungent, green, vegetable, fresh, fruity, fatty ^2^	18829-55-5	773	804	□	□	□	□	□	□	■	■	■	□	□	□
2,4-Heptadienal *	Fried, nut, fat, green, pungent, fruity, spicy ^1,2^	5910-85-0	852	865	□	□	□	□	□	□	■	■	■	■	■	■
Heptanal	Fat, citrus, rancid, fresh, aldehydic, green, herbal, wine-lee, ozone ^1,2^	111-71-7	909	917	■	□	■	■	■	■	■	■	■	■	■	■
Octanal	Citrus, soap, lemon, herbal, green, honey ^1,2^	124-13-0	877	907	■	■	■	■	■	■	■	■	■	■	■	■
Nonanal *	Fat, citrus, fresh, orange, green ^1,2^	124-19-6	929	931	■	□	■	■	■	■	■	■	■	■	■	■
Safranal *	Herb, sweet, fresh, phenolic, metallic, rosemary, tobacco, spicy ^1,2^	116-26-7	889	899	□	□	□	■	■	■	■	■	■	■	■	■
β-cyclocitral	Saffron, green, rose, sweet, tobacco, damascene, fruity, mint ^1,2^	432-25-7	843	850	□	□	□	□	□	□	■	■	■	□	□	□
Undecanal	Waxy, soapy, floral, aldehydic, citrus, green, fatty, fresh, laundry ^2^	112-44-7	72	865	□	□	□	□	□	■	□	□	□	□	□	□
Dodecanal	Soapy, waxy, aldehydic, citrus, green, floral ^2^	112-54-9	917	963	■	■	■	■	■	■	□	■	■	■	■	■
Tridecanal	Flower, sweet, must, fresh, clean, aldehydic, soapy, citrus, petal, waxy, grapefruit peel ^1,2^	10486-19-8	781	885	□	□	□	□	□	■	□	□	□	□	□	□
Tetradecanal	Fatty, waxy, amber, incense, dry, citrus, peel, musk ^2^	124-25-4	763	896	■	■	■	■	■	■	□	■	■	□	□	□
Pentadecanal *	Fresh, waxy ^1,2^	2765-11-9	823	956	□	■	■	■	■	■	■	■	■	■	■	■
**Alcohols**																
Ethanol *	Sweet^1^	64-17-5	976	976	■	□	■	■	■	■	■	■	■	■	■	■
2-Methyl-1-butanol	Malt, wine, onion, ethereal, fusel, alcoholic, fatty, greasy, whiskey, leathery, cocoa ^1,2^	137-32-6	855	876	□	□	□	□	□	□	■	■	■	■	■	■
(S)-(-)-2-Methyl-1-butanol	Ethereal, fresh ^2^	1565-80-6	796	847	□	■	■	■	■	■	□	□	□	□	□	□
3-Methyl-1-butanol/ isoamyl alcohol *	Whiskey, malt, burnt, fusel, oil, alcoholic, fruity, banana ^1,2^	123-51-3	877	894	■	□	■	■	■	■	□	□	■	■	■	■
1-Penten-3-ol	Ethereal, horseradish, green radish, chrysanthemum, vegetable, tropical fruity, truffle, oily, resinous ^2^	616-25-1	846	846	□	□	□	□	□	□	■	■	■	□	□	□
Z-3-Hexenol	Grass, fresh, green, cut, foliage, vegetable, herbal, oily ^1,2^	928-96-1	881	916	□	■	■	□	□	□	□	■	■	□	□	□
2-Ethylhexanol	Rose, green, citrus, fresh, floral, oily, sweet ^1,2^	104-76-7	930	943	■	■	■	■	■	■	■	■	■	■	■	■
(S)-2-Heptanol	Mushroom, oily, fatty, blue cheese, moldy ^2^	6033-23-4	800	859	□	□	□	□	■	■	□	□	□	□	□	□
3-Octenol	Mushroom, earthy, green, oily, fungal, raw chicken, vegetative ^2^	20125-85-3	852	878	□	□	□	□	□	□	■	■	■	□	□	□
Phenethyl alcohol *	Honey, spice, rose, lilac, floral, fresh ^1,2^	60-12-8	888	932	■	■	■	■	■	■	□	■	■	■	■	■
1-Dodecanol	Earthy, soapy, waxy, fatty, honey, coconut ^2^	112-53-8	799	896	□	□	□	□	□	□	■	■	■	□	□	□
Eugenol	Clove, honey, sweet, spicy, clove, woody ^1,2^	97-53-0	864	868	□	□	□	■	■	■	■	■	■	■	■	■
Cedrol *	Cedarwood, woody, dry, sweet, soft ^2^	77-53-2	661	825	□	■	■	■	■	■	□	■	■	■	■	■
**Ketones**																
Geranyl acetone *	Magnolia, green, fresh, fruity, waxy, rose, woody, tropical ^1,2^	3796-70-1	682	777	□	□	□	□	■	■	■	■	■	■	■	■
2,3-Butanedione	Strong, butter, sweet, creamy, pungent, caramel, milky ^2^	431-03-8	894	894	□	□	□	□	□	□	■	■	■	□	□	□
2-Octanone	Earthy, weedy, natural, woody, herbal, dairy, waxy, cheese, woody, mushroom, yeast ^2^	111-13-7	842	842	□	□	□	□	□	□	■	■	■	□	□	□
3,5-Octadien-2-one *	Fruity, fatty, mushroom ^2^	38284-27-4	879	895	□	■	■	■	■	■	■	■	■	■	■	■
Isophorone	Cooling, woody, sweet, green, camphor, fruity, musty, cedarwood, tobacco, leather ^2^	78-59-1	699	773	□	■	■	□	□	□	■	■	■	□	□	□
Ketoisophorone	Musty, woody, sweet, tea, tobacco, leaf, citrus, floral, musty, tea like with green, sweet, fruity nuances ^2^	1125-21-9	808	808	□	□	□	□	□	□	■	■	■	□	□	□
2,2,6-Trimethylcyclohexanone	Pungent, thujonic, labdanum, honey, cistus ^2^	2408-37-9	729	729	□	□	□	□	□	□	■	■	■	□	□	□
(−)-Carvone	Mint, sweet, spearmint, herbal ^1,2^	6485-40-1	918	921	□	■	■	■	■	■	□	■	■	■	■	■
Benzophenone	Balsamic, herbal, rose, metallic, Geranium ^2^	119-61-9	623	696	□	□	□	□	□	□	■	■	■	□	□	□
β-Damascenone *	Apple, rose, honey, tobacco, sweet ^1,2^	23726-93-4	898	955	■	□	■	■	■	■	■	■	■	■	■	■
Dihydro-β-ionone	Earthy, woody, mahogany, orris, dry amber ^2^	17283-81-7	768	768	□	□	□	□	□	□	■	■	■	□	□	□
Megastigmatrienone	Sweet, nutty, skin, tobacco, spicy^2^	38818-55-2	815	822	□	□	□	□	□	□	■	■	■	□	□	□
**Monoterpenes**																
3-δ-Carene *	Citrus, terpenic, herbal, pine, solvent, resinous, phenolic, cypress, medicinal, woody ^2^	13466-78-9	741	825	□	■	■	□	□	□	□	■	■	□	□	□
Myrcene *	Balsamic, must, spice, peppery, terpene, plastic ^1,2^	123-35-3	834	892	□	■	■	■	□	□	□	■	■	□	□	□
Menthone	Fresh, green, minty ^1,2^	89-80-5	844	859	□	□	□	■	□	□	□	□	□	□	□	□
Camphene *	Camphor, woody, herbal, fir, needle, camphor, terpenic ^1,2^	79-92-5	815	860	□	■	■	■	■	■	□	■	■	■	■	■
α-Terpineol	Oil, anise, mint, lemon, citrus, ^1,2^	98-55-5	901	921	□	■	■	■	■	■	□	■	■	■	■	■
γ-Terpineol	Terpineol, lilac ^2^	586-81-2	951	956	■	□	■	■	■	■	□	□	□	□	□	□
1,5,8-*p*-Mentatriene	Roasted ^2^	21195-59-5	735	780	□	□	□	□	□	□	■	■	■	□	□	□
α-Ionone *	Wood, violet, sweet, floral, orris, tropical, fruity ^1,2^	127-41-3	892	897	□	■	■	■	■	■	■	■	■	■	■	■
β-Ionone *	Seaweed, violet, flower, raspberry, woody, sweet, fruity, berry, tropical, beeswax ^1,2^	14901-07-6	847	849	■	■	■	■	■	■	■	■	■	■	■	■
**Acids**																
Acetic acid *	Acidic, sour, pungent, vinegar ^1,2^	64-19-7	943	956	■	■	■	■	■	■	□	■	■	■	■	■
2-methylbutanoic acid	Pungent, acid, Roquefort, cheese ^2^	64-19-7	943	956	■	■	■	■	■	■	□	■	■	■	■	■
Isovaleric acid *	Sweat, acid, rancid, sour, stinky, feet, cheese, tropical ^1,2^	503-74-2	818	852	■	□	■	■	■	■	□	□	■	■	■	■
Caproic acid *	Sweat, sour, fatty, cheese ^1,2^	142-62-1	897	920	■	□	■	■	■	■	□	□	■	■	■	■
Octanoic acid *	Acid, sweat, cheese, fruit notes ^1,2^	124-07-2	921	934	■	□	■	■	■	■	□	□	■	■	■	■
Nonanoic acid *	Green, fat, waxy, dirty, cheese, cultured, dairy ^1,2^	112-05-0	887	900	■	■	■	■	■	■	□	■	■	■	■	■
Decanoic acid *	Rancid, fat, unpleasant, rancid, sour, fatty, citrus ^1,2^	334-48-5	924	933	■	□	■	■	■	■	□	□	■	■	■	■
Lauric acid	Metal, mild, fatty, coconut, bay, oil ^1,2^	143-07-7	689	826	■	□	■	■	■	■	□	□	■	□	□	□
**Monoterpenes alcohol**																
1-Terpinen-4-ol	Turpentine, nutmeg, must, pepper, woody, earth, musty, sweet ^1,2^	562-74-3	860	898	□	■	■	□	□	□	□	■	■	□	□	□
Linalool *	Citrus, flower, lavender, sweet, green ^1,2^	78-70-6	942	945	■	■	■	■	■	■	■	■	■	■	■	■
Linalool oxide	Flower, wood, musty, camphor, fenchyl, alcohol ^1,2^	60047-17-8	900	928	■	■	■	■	■	■	■	■	■	■	■	■
Trans-Linalool oxide *	Flower ^1,2^	34995-77-2	880	892	□	■	■	□	□	□	■	■	■	■	□	□
Cis-Linalool oxide	Earthy, floral, sweet, woody ^1,2^	5989-33-3	838	852	□	□	□	□	■	□	■	■	■	■	□	□
**Furans**																
Furfural	Bread, almond, sweet, woody ^1,2^	98-01-1	916	945	□	□	□	□	□	□	■	■	■	□	□	□
5-methylfurfural	Almond, caramel, burnt sugar, spice, maple, sweet, brown, grain, maple-like ^1,2^	620-02-0	884	911	□	□	□	□	□	□	■	■	■	□	□	□
2-Ethylfuran	Chemical, beany, ethereal, cocoa, bready, malty, coffee, nutty, brown, rooty, earthy, musty ^2^	3208-16-0	626	766	□	□	□	□	□	□	■	■	■	□	□	□
Furfuryl alcohol	Burnt, alcoholic, chemical, musty, sweet, caramel, bread, coffee ^2^	98-00-0	806	849	■	□	■	□	□	□	□	□	□	□	□	□
(S)-dihydroactinidio-lide *	Musk, coumarin ^2^	17092-92-1	888	905	■	■	■	■	■	■	■	■	■	■	■	■
**Phenols**																
4-Ethylphenol	Phenolic, castoreum, smoke, guaiacol ^2^	123-07-9	817	856	■	□	■	■	■	■	□	□	■	■	■	■
4-Ethylguaiacol	Spice, clove, smoky, bacon, phenolic ^1,2^	2785-89-9	908	939	■	□	■	■	■	■	□	□	■	■	■	■
**Pyrrol**																
1-Furfurylpyrrole	Plastic, green, waxy, fruity, coffee, vegetable, vegetative, onion, sharp and metallic ^2^	1438-94-4	859	859	□	□	□	□	□	□	■	■	■	□	□	□
**Heterocyclic aromatic organic compound**																
4,5-Dimethyl-2-propyloxazole	Roasted, burnt ^2^	53833-32-2	674	674	□	□	□	□	□	□	■	■	■	□	□	□

Note: K: kombucha; BT K starter: black tea kombucha starter; CL: coffee leaf; CL-TM: coffee-leaf-toasted maté; TM: toasted maté; Inf: infusion; d0, d3, d6, and d9: 0, 3, 6, and 9 days of fermentation. ^a^ CAS# (Chemical Abstracts Service) Registry Number, available in the NIST database [99]; ^b^ ELRI: Experimental Linear Retention Index; ^c^ LRI: Linear Retention Index based on the literature and the NIST database [99]; ^1^ http://www.flavornet.org [100]; ^2^ http://www.thegoodscentscompany.com [101]; * Impact compounds according to the literature: Steger et al. [13]; Ubeda et al. [71]; Tran et al. [72]; Bishop et al. [73]; Wang et al. [80]; Wang et al. [81]; Araújo et al. [85]; Marquez et al. [86]; Mei et al. [87]; Machado et al. [102]; Kang et al. [103]; Procopio et al. [104]; ■ compound identified in the sample; □ compound not identified in the sample.

Aldehydes usually have a low threshold in foods and contribute to their overall flavors [76]. Pentadecanal, benzaldehyde, octanal, and hexanal, identified in CL K and CL-TM K, are key odorants in coffee leaf tea [13,87]. Additionally, octanal and nonanal were previously identified in green maté [105].

In CL-TM K, the aldehyde 2,4-heptadienal is a marker compound in green and toasted maté [85,102]. The formation of this VOC is favored by some steps in the maté processing such as scorching and drying, where the material is subjected to heat treatment [86]. According to Mei et al. [87], 2,4-heptadienal is an impact compound in coffee leaf, although we did not identify it in CL infusion or CL K.

Regarding alcohols, 2-methyl-1-butanol, identified only in CL K samples, was identified in a green coffee spirit [106]. Ethanol, identified in all kombucha samples, can impact the aromatic profile of kombucha [72]. Additional alcohol identified was isoamyl alcohol, one of the most important sensory-active higher alcohols for beer aroma [104]. Regarding acids, nonanoic acid is an odor active compound in coffee leaf tea [13] and was identified in CL K and CL-TM K. Acetic acid is the main acid responsible for kombucha sourness [72,73]. Decanoic and octanoic acid were identified in all CL K and CL-TM K. They are reported as odor-active compounds in sparkling wine [71].

In kombuchas, ethyl acetate and methyl salicylate were identified in all CL Ks and CL-TM Ks; methyl salicylate is an odor-active compound in coffee leaf tea [87], Pu-erh tea [81], and oolong tea [107] and was previously identified in green maté [103]. Ethyl acetate imparted apple and banana traits to wine [108], while ethyl decanoate and ethyl octanoate have been reported as abundant compounds in ciders [109]; ethyl isobutyrate and ethyl hexanoate have been reported as impact compounds in sparkling wine [71]; ethyl phenylacetate is one of the important esters in wine aroma compounds formed during alcoholic fermentation [108]; and ethyl laurate was correlated with positive aroma compounds in beer [110].

According to Steger et al. [13], 3,5-octadien-2-one, α-ionone, and β-ionone are key odorants in coffee leaf tea fermented by yeasts. These compounds were identified in CL infusion prior to kombucha preparation. Additional compounds identified in CL K were menthone and carvone, important contributors to Pu-erh tea aroma [81]. Also, β-damascenone, α-ionone, and β-ionone are odor-active compounds in green maté [86]. Geranyl acetone was previously identified as a marker compound in green maté [85,86,102]. α-terpineol was previously identified in coffee leaf and green maté teas [13,14,87,111]; linalool and linalool oxide are odor active compounds in green maté [86].

The only furan identified in all infusions and kombucha samples was dihydroactinidiolide. This VOC is viewed as critical in determining the aroma characteristics of black tea [96] and Pu-erh tea [81]. It has been previously identified in coffee leaf tea [14] and green maté [85]. This VOC can be generated by photo-oxidation of β-carotene under UV light [96]. The presence of the phenols 4-ethylguiacol and 4-ethylphenol in BT K, CL K, and CL-TM K is probably due to the fermentative process by yeasts from the genus *Brettanomyces* through conversion of hydroxycinnamic acids [112,113].

### 3.4. Sensory Tests

#### 3.4.1. Acceptance Test

**Figure 4:**
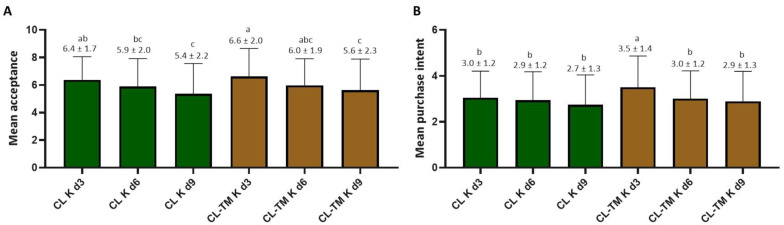
Mean acceptance scores (**A**) and mean purchase intent scores (**B**) of coffee leaf (CL K) and coffee leaf-toasted maté (CL-TM K) kombuchas by Rio de Janeiro consumers (*n* = 103). Different letters over the bars indicate significant difference (at *p* < 0.05) by ANOVA followed by the Tukey test. Note: d0, d3, d6, and d9: 0, 3, 6, and 9 days of fermentation.

The consumer assessors’ main characteristics are presented in Table 3. After exclusions, a total of 103 assessors participated in the sensory assessment. The mean acceptance scores for CL K d3, d6, and d9 were 6.4, 5.9, and 5.4, respectively (Figure 4). Considering that in our previous study [14], blending with toasted maté tea increased the acceptance of CL infusions, we chose to blend CL and TM, aiming to increase the acceptability of CL- K. Acceptance of CL-TM K d3, d6, and d9 were 6.6, 6.2, and 5.9, respectively (Figure 4). Therefore, the highest mean scores were given to CL-TM K d3 and CL K d3, with 75% and 77%, of the scores, respectively, between six and nine. When scoring the samples, the assessors could comment on them if they wished. The most cited descriptors were ‘sweet’ for CL K and CL-TM K samples on d3 and d6 and ‘taste of toasted maté’ for CL-TM K. Additional descriptors cited by assessors for CL K and CL-TM K were soft drink, peach, and peach syrup and bitter (consumers often tend to confuse acidity and bitterness sensations).

The preference for higher sweetness was caused by the need to balance the acidity caused by the increased number and abundance of the organic acids during fermentation. The preference of Brazilians for sweeter foods and the higher soluble solids in d3 content were also likely responsible for this result.

According to Meilgaard et al. [41], for a sample to be considered “well-accepted,” it must obtain a 70% Acceptance Index (AI) or higher. Only the two most-accepted samples had higher AI, while the AI for d6 was 67%. The higher acidity was the main reason for the low score given on d9 (AI = 61%, on average). However, in countries where food is less sweet, like in Europe and perhaps in the U.S., d9 might have been better accepted, given that sugar consumption in these countries has decreased considerably over the last years [114,115]. As usual, the purchase intent results were associated with those from the acceptance test (Figure 4A).

The high acceptance mean for CL-TM K may be explained by food pairing. The “food pairing hypothesis” states that two ingredients that share chemical compounds are more likely to taste (and smell) good together [116]. In the present study, the volatile composition of CL and CL-TM infusions and kombuchas were similar (Table 2). Such similarity has also been observed for infusions in our previous study [14] in which the addition of toasted maté tea increased consumers’ acceptance of coffee leaf tea. These two plants also share many non-volatile compounds, including the type and content of polyphenols and methylxanthines [28,117].

Making kombucha from coffee leaf infusions increased its acceptability, as compared to data from DePaula et al. [14] using the same raw materials. The referred study obtained 6.1 as a mean score for coffee leaf tea and 6.3 when a blend with 50% toasted maté was tested. No study using coffee leaf tea as a substrate for kombucha production was found for comparison with the present results. However, a study from the South of Brazil by Dartora et al. [118] conducted a sensory analysis of black tea, green tea, and green maté kombucha prepared with 5% (*w*/*v*) sugar, 0.5% (*w*/*v*) green maté and a SCOBY composed mainly by *Brettanomyces bruxellensis* and *Komagaeitabacter rhaeticus* as the major yeast and bacteria, respectively. Green maté kombucha presented a higher acceptance mean score (6.2) than black tea (5.8) or green tea (5.7) kombuchas. Assessors also expressed good feelings and sensations with emojis for green maté, while for black and green tea kombucha, the emojis were used to express negative feelings. It is worth noting that green maté is largely consumed in the South region of Brazil [118].

In the study conducted by Ulusoy and Tamer [45] in Turkey, the sensory acceptance of kombuchas made from new substrates such as black carrot, cherry laurel, blackthorn, and red raspberry and prepared with 6% *w*/*v* of sugar and a similar SCOBY, was evaluated using a nine-point hedonic scale. The beverages fermented for shorter periods (3 and 5 days) received scores between six and eight, while those fermented for 10 and 12 days received scores below five. The authors state that products with ratings below the five-point limit value are unlikely to be commercially successful. Moreover, studies conducted in Brazil and Tunisia have shown that assessors enjoyed herbal and grape kombuchas after 6 days of fermentation, with average acceptance scores ranging from five to seven. [119,120].

Cluster analysis was performed to identify different consumer niches. Cluster 1 (*n* = 41, mean score for all beverages = 6.9) consistently attributed the highest scores to CL-TM K 3d (acceptance = 7.7, AI = 86%). This cluster was composed of 54% female; 43% were between 18–24 years old, 41% had incomplete graduations, and 36% had family monthly incomes of 2–3 MW. In this cluster, 12% of the assessors were kombucha consumers. This cluster had the highest consumption of soda (71%), sparkling water (46%), sparkling wine (34%), tonic water (24%), cider (20%), and apple juice (15%),

Cluster 2 (*n* = 30, mean score considering all beverages = 5.1) also consistently attributed the highest scores to CL-TM K 3d (acceptance mean = 7.6, AI = 84%). This cluster was 77% female; 63% were between 18–24 years old and had incomplete graduations, and 33% had family monthly incomes of 2–3 MW. In this cluster, 16% of the assessors were kombucha consumers. Samples with low fermentation time were more accepted by the assessors (*p* < 0.0001). This cluster had the highest consumption of soda (50%) and the lowest consumption of sparkling wine (37%), sparkling water (30%), cider (17%), tonic water (13%), and apple juice (7%) as compared to cluster 1. The present results reinforce the fact that coffee leaf blends with toasted maté are well-accepted because toasted maté is largely consumed in the southeast of Brazil, especially in Rio de Janeiro [14,15,117].

In Cluster 3 (*n* = 32, mean acceptance score considering all samples = 5.5), most assessors attributed the highest scores to CL K 3d (mean acceptance score= 6.5; AI = 72%). This cluster was 78% female; 56% were between 18–24 years old; 46% had incomplete graduations, and 38% had family monthly incomes of 2–3 MW. In this cluster, 16% of the assessors were kombucha consumers. This cluster had a high consumption of soda (63%) and sparkling water (50%), followed by tonic water (34%), sparkling wine (31%), apple juice (19%), and cider (13%).

It is worth mentioning that the young age assessors were naturally selected by their will for participating in the study. The type of people willing to participate in a study that offers no reward other than the beverage itself indicates the inclination to consume the product. The study findings suggest that young adults are potential consumers of pure and blended CL K with a low fermentation period. In the questionnaire, it was clear that those who frequently consumed soda, a sparkling sweet beverage, were likely to attribute higher scores to kombuchas fermented for 3 days only (*p* < 0.0001), the samples with the highest amount of sucrose (Table 1). It is possible that adding CO_2_ to the beverage will increase acceptance due to the increased resemblance to soda. Considering that the Brazilian consumers show preferences for sweeter beverages, especially among young consumers, d9 samples received low mean acceptance and purchase intent scores. Kombucha is a new beverage in Brazil, and understanding the consumers’ and non-consumers’ sensory perception toward the product brings essential information for the market to launch new products more accurately and assertively [118].

It is worth mentioning that while commercial kombuchas generally receive additives to increase flavor intensity and variety [121], in the present study, the kombuchas were naturally flavored, that is, with no additions of fruits, herbs, or spices to flavor them.

#### 3.4.2. Rate All That Apply (RATA)

**Figure 5:**
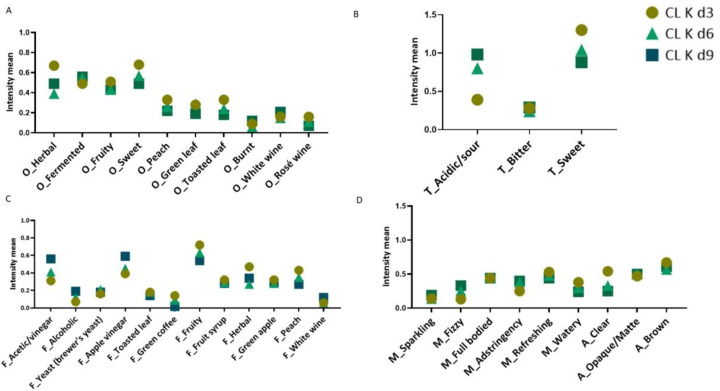
Intensity means for odor (**A**), taste (**B**), flavor (**C**), and mouthfeel and appearance (**D**) for coffee leaf kombuchas (CL K) with 3, 6, and 9 days of fermentation (3d, 6d, and 9d). Note: O: odor; T: taste; F: flavor; M: mouthfeel; A: appearance. d0, d3, d6, and d9: 0, 3, 6, and 9 days of fermentation.

**Figure 6:**
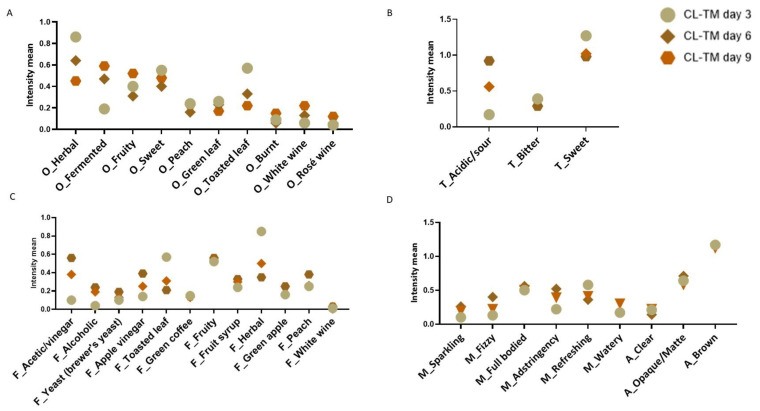
Intensity means for odor (**A**), taste (**B**), flavor (**C**), and mouthfeel and appearance (**D**) for CL-TM kombuchas with 3, 6 and 9 days of fermentation. Note: O: odor; T: taste; F: flavor; M: mouthfeel; A: appearance; CL-TM: coffee leaves with toasted maté. d0, d3, d6, and d9: 0, 3, 6, and 9 days of fermentation.

A RATA test was performed to identify changes during the fermentation of CL K (Figure 5) and CL-TM K (Figure 6). The fermentation time caused differences in the intensity of the sensory descriptors (*p* < 0.0001), while the presence of toasted maté in coffee leaf kombucha did not cause statistical differences.

On d3, CL K presented higher intensities for herbal and sweet odors; sweet taste; fruity, herbal, and peach flavors; and clear appearance (Figure 5). This was also observed by Steger et al. [13], who observed that fermented coffee leaf infusions tended to produce sweetish fruity notes, especially a peach-like aroma and flavor. As expected, due to the microbial activity during fermentation, on d6, while the herbal, fruity, and peach flavors and sweet odor and taste decreased, the intensity of the fermented odor increased together with an acidic/sour taste and acetic/vinegar and apple vinegar flavors. CL K d9 presented the low intensity mean mainly for sweet odor and taste and the highest intensity for acidic/sour and acetic/vinegar tastes, apple vinegar flavors, and a fizzy mouthfeel.

The descriptors with the highest intensity means attributed to CL-TM K d3 (Figure 6) were the following: herbal, toasted leaf, fruity and sweet aromas, sweet taste, toasted leaf, herbal flavor, and refreshing. Similar descriptors have been obtained for toasted maté in our previous study [14]. Higher intensity mean for fermented aroma and an acidic/sour taste and an acetic/vinegar flavor were attributed to CL-TM K d6 and d9, while herbal and toasted leaf odors and sweet taste intensities decreased. CL-TM K also showed a higher frequency of herbal descriptors for aroma and flavor, as observed by DePaula et al. [14], for blended coffee leaf and toasted maté infusions.

Some specific aromas and flavors perceived in kombuchas by the assessors (peach, white, and rosé wine aromas and green coffee, fruit syrup, green apple, peach, and white wine flavors) were not identified in the infusions in our previous study using the same raw materials [14]. This result ratifies the change in the sensory profiles and the increases in flavor complexities caused by fermentation by the SCOBY microorganisms. Higher intensities for sweet aromas and tastes on d3 and d6 can be attributed to high soluble solids contents. In comparison, higher intensity means that the acid/sour tastes on d9 are attributed to the higher organic acid concentration reflected in higher TA and lower pH values [121].

**Figure 7:**
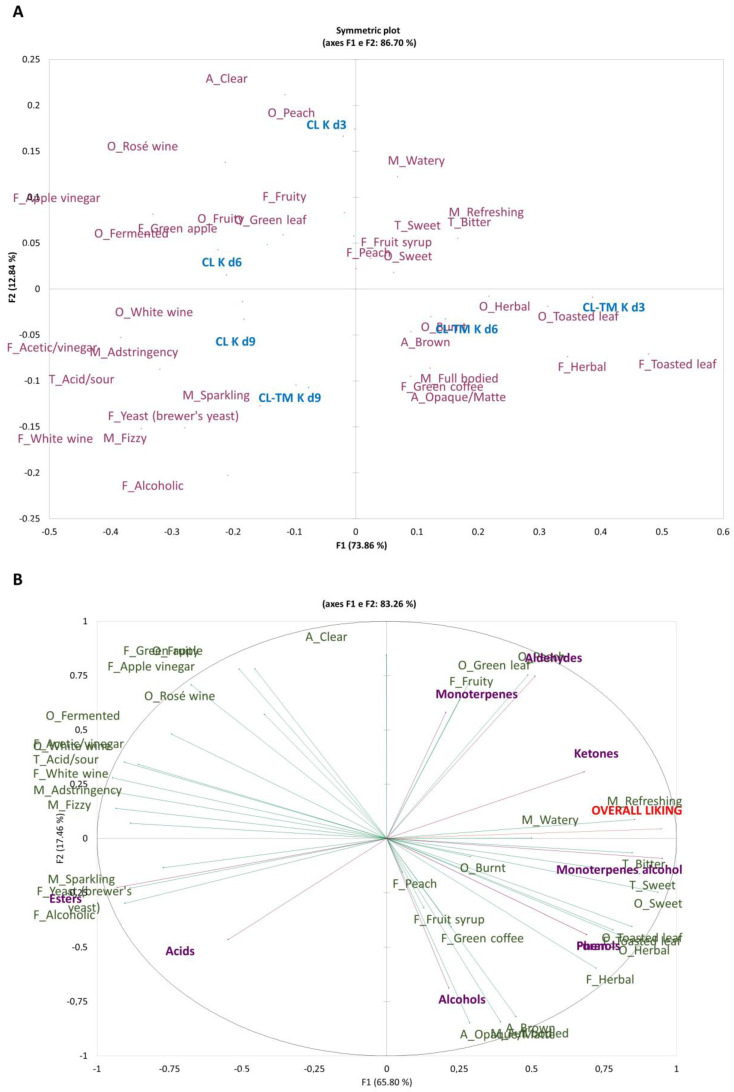
Correspondence analysis (CA): bi-dimensional plot of the samples of coffee leaf (CL K) and coffee-toasted maté leaf (CL-TM K) kombuchas after 3, 6, and 9 days of fermentation (d3, d6, and d9) (**A**) and sensory descriptors attributed by consumer assessors (*n* = 103) through the RATA test, distributing volatile compounds and descriptors that make up the best acceptance of samples among consumers (**B**). Overall liking and the volatile compounds were considered to be supplementary variables. Note: O: odor; T: taste; F: flavor; M: mouthfeel; A: appearance.

Correspondence Analysis (CA) was applied to the RATA descriptors to generate the sensory map shown in Figure 7A. The first and second dimensions of the map explained 73.86% and 12.84% of the experimental data variance, respectively, representing 86.70% of the total variance (*p* < 0.0001). It is possible to see similarities between CL K on d3 and d6, between CL K on d9 and between CL-TM K on d9 and CL-TM K on d3 and d6 because the same descriptors were used to best describe these samples.

Figure 7B presents the main sensory descriptors reported for the individual samples in the RATA test by the assessors in association with the acceptance scores and classes of volatile compounds used as secondary variables. The first two dimensions explained 83.26% of the variability, with 65.80% of the variance explained by dimension 1 and 17.46% by dimension 2. The descriptors leading to higher acceptance rates were those used to describe the samples at days 3 and 6 of fermentation with a higher intensity mean (Figure 5 and Figure 6), which are associated with fruity, sweet, and herbal descriptors. Although phenols can impart undesirable odors to wine [112], they seem not to impact kombucha aromas because their area in chromatograms was lower than ketones, monoterpenes, and monoterpenes alcohols (Figure 3).

It is worth noting that the distribution of chemical classes in Figure 7B only considered the number of volatile compounds in each chemical class, together with the descriptors obtained in the RATA test. It did not consider the chromatogram peak areas or the odor threshold of the compounds. Nevertheless, the distribution of the classes is reasonably similar to the odor and flavor descriptions in the literature, which can be revisited in Table 2. This type of distribution seems to work better when a food matrix has a high number of volatile compounds of a certain class, given that a small number of important potential impact compounds could be neglected [14]. Table 4 contains the RATA and related aroma and flavor descriptors related with the volatile compounds identified.

**Figure 8:**
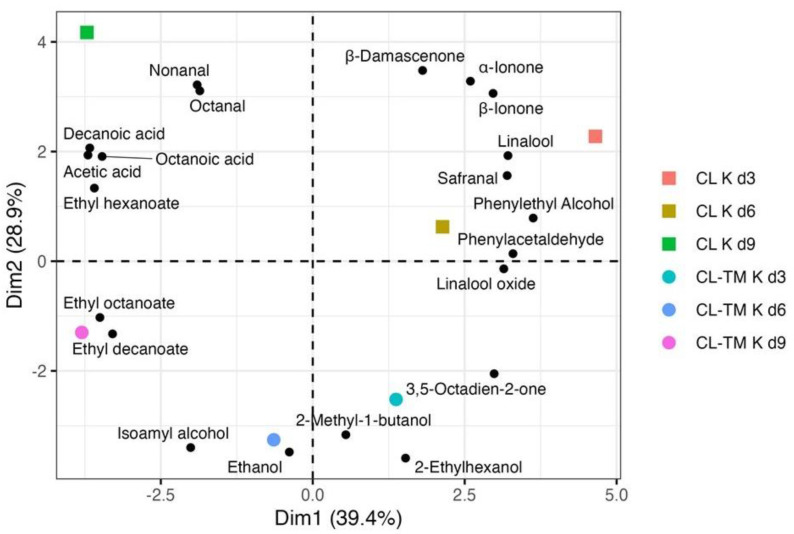
Biplot of the principal component analysis (PCA) of the volatile compounds relevant for RATA attributes, as summarized in Table 4. Coffee leaf (CL K) and coffee-toasted maté leaf (CL-TM K) kombuchas after 3, 6, and 9 days of fermentation (d3, d6, and d9).

The principal component analysis associated the volatile compounds profile withRATA aroma and flavor descriptors. The biplot obtained (Figure 8) highlights that the changes on the profile of these selected volatiles during the kombucha fermentation are in agreement with those observed in the sensory characteristics. The observed changes in sensory characteristics during kombucha fermentation align with the evolution of the profiles of the selected volatiles. Similar changes in the profiles of the selected volatiles were noted in both CL K and CL-TM K from d3 to d9, although differences were observed between the two, as also noted in the RATA test. The PCA biplot explains the higher intensity of fruit, sweet, herbal, and peach flavors on d3 (associated with 3,5-octadien-2-one, α-ionone, phenylacetaldehyde, phenylethyl alcohol, linalool, β-damascenone, safranal, and linalool) and the impact of microbial activity on d6 and d9 that promoted the highest intensity for acidic/sour taste and acetic/vinegar, apple vinegar flavors, and fizzy mouthfeel (linked with nonanal, octanoic acid, decanoic acid, ethyl hexanoate, ethyl octanoate, ethyl decanoate, β-damascenone, linalool, and isoamyl alcohol).

## 4. Final Considerations and Conclusions

In the present study, a total of 100 volatile organic compounds were identified after considering all infusions and kombucha samples: 36 in the black tea kombucha starter, 75 in coffee leaf kombuchas, and 90 in coffee-leaf-toasted maté kombuchas. Coffee leaf and coffee-leaf-toasted maté kombuchas presented similar volatile profiles. Thirty potential impact compounds were common to them (ethyl hexanoate, ethyl isobutyrate, isoamyl acetate, methyl salicylate, benzaldehyde, hexanal, nonanal, pentadecanal, safranal, phenethyl alcohol, isoamyl alcohol, cedrol, ethanol, 3,5-Octadien-2-one, geranyl acetone, β-damascenone, 3-δ-Carene, myrcene, camphene,α-ionone,β-ionone, acetic acid, caproic acid, decanoic acid, isovaleric acid, nonanoic acid, octanoic acid, linalool, trans-linalool oxide, and (S)-dihydroactinidiolide), while only one impact compound was exclusive of kombuchas containing toasted maté (2,4-heptadienal).

The microbial consortia (SCOBY) used in this study were represented mostly by the acetic acid bacteria and yeasts from the genera *Komagateibacter* and *Pichia*, respectively. *Komagataeibacter rhaeticus* and *Saccharomyces cerevisiae* were more abundant in coffee-leaf-toasted mate kombuchas than in coffee leaf kombuchas, while the contrary was true of *Pichia* sp. These differences indicate that raw materials can change the initial SCOBY profile during fermentation. This result is ratified by differences observed between the microbial profile found in this study and the one found in coffee cascara kombucha using the same initial starter culture [35]. More studies are needed to verify the contribution of each food matrix to the SCOBY profile, and the impact of these differences on the aroma, flavor, and bioactivity of the resulting kombuchas.

Coffee leaf kombuchas and coffee-leaf-toasted maté kombuchas, especially those containing higher sucrose content and lower acidity, were accepted by Rio de Janeiro consumers. With herbal, toasted leaves, fruity and sweet traits, and lower acidity, coffee-toasted maté leaf kombuchas were only slightly more accepted than coffee leaf kombuchas. The authors believe that toasting coffee leaves would have increased acceptance by these assessors given the chemical resemblance to toasted maté. The volatile composition of the beverages supported the sensory characterization of kombucha samples.

Most people who agreed to participate in the study (53%) were aged 18–24 years. These were also the people who most regularly consumed similar beverages to kombucha, like soda, sparkling water, and sparkling wine. It has been estimated that the young people in Latin and North America will shape the market for the next decades [134]. Considering that they are looking for natural soft drinks that are perceived as promoting well-being and have a limited amount of added sugar, kombuchas and sparkling kombuchas prepared with pure or blended coffee leaf with other herbs or teas could be of great interest to them, given that these leaves are rich in bioactive compounds and have exerted health-functional properties in vitro [10], with limited amount of caffeine for those who are sensitive to its effect.

In conclusion, coffee leaf was shown to be a suitable raw material for producing aromatic, natural, and potentially healthy kombucha beverages. Given the fact that Brazil is responsible for one-third of the world’s coffee production, the acceptance of coffee leaf kombucha by Brazilians opens a large perspective for the national coffee growers and food industry. Concomitantly, this is a way to reduce the environmental pollution caused by incorrect disposal after harvest season and the pruning of coffee trees, improving the coffee chain value and allowing sustainability to be aligned with the United Nations and FAO goals for 2030. Giving the numerous known health benefits of coffee consumption, the similar chemical composition of the leaf in many aspects, and the potential benefits of fermentation for increasing the bioaccessibility of polyphenols and other components of the beverage, the health effects of usual coffee leaf kombucha drinking should be evaluated in future studies.

## Figures and Tables

**Table 1:** Physicochemical characteristics of coffee leaf and coffee-leaf-toasted maté kombuchas.

Samples	Days of Fermentation	Titrable Acidity (mEq/L)	pH	Soluble Solids (°Brix)	Sucrose (g/100 mL)
CL	0	0.1 ± 0.04 ^a^	3.9 ± 0.21 ^a^	12.2 ± 0.00 ^a^	11.1 ± 1.06 ^a^
	3	0.3 ± 0.06 ^b^	3.8 ± 0.14 ^a^	9.7 ± 0.28 ^b^	8.8 ± 1.06 ^b^
	6	0.8 ± 0.00 ^c^	3.6 ± 0.07 ^a^	9.4 ± 0.07 ^b^	8.4 ± 1.06 ^b^
	9	0.9 ± 0.10 ^c^	3.4 ± 0.07 ^b^	9.2 ± 0.07 ^b^	7.0 ± 1.06 ^c^
CL-TM	0	0.1 ± 0.04 ^a^	4.0 ± 0.00 ^a^	11.8 ± 0.91 ^a^	10.7 ± 1.06 ^a^
	3	0.1 ± 0.04 ^a^	4.0 ± 0.00 ^a^	9.9 ± 0.28 ^a^	10.2 ± 1.06 ^a^
	6	0.2 ± 0.05 ^a^	3.9 ± 0.01 ^a^	9.6 ± 0.28 ^a^	9.1 ± 1.06 ^b^
	9	0.3 ± 0.06 ^b^	3.9 ± 0.01 ^a^	9.1 ± 0.91 ^a^	7.2 ± 1.06 ^c^

Data are expressed as mean ± standard deviation (*n* = 3); different superscript letters on the same column for the same beverage indicate significant difference (at *p* < 0.05) by ANOVA followed by the Tukey test; CL: coffee leaf tea; CL-TM: blend of coffee leaf tea and toasted maté tea.

**Table 3:** Socio-economic profiles of the sensory tests assessors.

Gender		Age				
Male	Female	18–24	25–34	34–44	45–59	≥56
32%	68%	53%	32%	6%	4%	5%
**Level of education**						
Basic education	Undergraduated	Incomplete graduation	Complete graduation		Master’s or doctoral degree	
2%	48%	15%	12%	24%		
**Family income (MW: minimum wages)**						
1 MW	2–3 MW	4–5 MW	>5 MW			
22%	37%	19%	22%			
**Know kombucha**		**Drink kombucha**				
Yes	No	Yes	No			
64%	36%	16%	84%			
**Sparkling beverages/soft drinks consumption**						
Sparkling water	Apple juice	Soda	Tonic water	Sparkling wine		Cider
44%	13%	62%	24%	34%		17%

**Table 4:** RATA aroma and flavor descriptors for coffee leaf and coffee leaf-toasted maté kombuchas and the corresponding volatile compounds identified in the present study (from Table 2).

RATA and Related Aroma and Flavor Descriptors	Corresponding Volatile Compounds	References
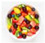 Fruity	3,5-octadien-2-one, α-ionone, geranyl acetone, isophorone, hexanal, benzaldehyde, ethyl acetate	[13,14,55,87,122]
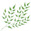 Herbal/Green leaf	Hexanal, heptanal, nonanal, octanal, Z-3-hexenol, phenylacetaldehyde, phenylethyl alcohol, linalool	[14,79,87]
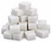 Sweet	Benzaldehyde, α-ionone, β-damascenone, safranal, linalool	[14,87]
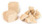 Fermented	Hexanal, benzaldehyde, nonanal, phenylacetaldehyde, ethanol, octanoic acid, isoamyl alcohol	[122]
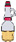 Vinegar	Acetic acid	[72]
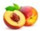 Peach	Benzaldehyde, hexanal, geranyl acetone, linalool, myrcene, eugenol, β-ionone, β-damascenone	[123,124]
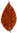 Toasted leaf	Furfural	[14]
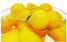 Fruit syrup	Ethyl octanoate, nonanal, linalool, benzaldehyde	[125]
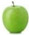 Green apple	2-methyl-1-butanol, hexanal, nonanal	[126]
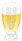 Yeast (Brewer’s yeast)	Nonanal, ethanol, octanoic acid, isoamyl alcohol, ethyl decanoate, ethyl hexanoate	[127]
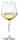 White wine	Ethyl acetate, ethyl hexanoate, ethyl octanoate, ethyl nonanoate, ethanol, acetic acid, decanoic acid, octanoic acid, benzaldehyde, β -damascenone	[128]
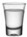 Alcoholic	Ethyl acetate, ethyl hexanoate, ethyl decanoate, linalool oxide, ethanol, isoamyl alcohol	[58,122,129]
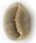 Green coffee	Acetic acid, isoamyl alcohol, 2-ethylhexanol, phenylethyl alcohol	[130,131]
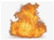 Burnt	2-methylbutanal; isoamyl alcohol	[100,132]
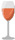 Rosé wine	Nonanal, 1-dodecanol, 1-heptanol, octanoic acid, decanoic acid, ethyl hexanoate, ethyl octanoate, ethyl decanoate, β-damascenone, linalool	[133]
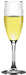 Sparkling wine	Hexanoic acid, octanoic acid, ethyl isobutyrate, ethyl hexanoate, isoamyl acetate	[71]

## Data Availability

The original contributions presented in the study are included in the article, further inquiries can be directed to the corresponding authors.
